# Anti-violence measures developed by ILO and WHO: Analysis of the prevalence of workplace violence and the effects of implementation in a general hospital in China

**DOI:** 10.3389/fpubh.2022.1049832

**Published:** 2022-12-14

**Authors:** Yubing Hu, Qianqian Luo, Rui Li, Min Zhang, Yuming Wang, Peipei Su, Jing Sun, Ying Li, Yujie Liu, Na Chen, Yiming Huang

**Affiliations:** ^1^Institute of Nursing and Health, Henan University, Kaifeng, China; ^2^Department of Nursing, The Third People's Hospital of Zhengzhou, Zhengzhou, China; ^3^School of Population Medicine and Public Health, Chinese Academy of Medical Sciences and Peking Union Medical College, Beijing, China; ^4^Scientific Research Division, Henan Provincial People's Hospital, Zhengzhou, China; ^5^Rheumatology Immunology Department, The First Affiliated Hospital of Henan University of CM, Zhengzhou, China; ^6^Department of Nursing, Central Hospital of Zhengzhou University, Zhengzhou, China; ^7^Out-patient Department, The First Affiliated Hospital of Zhengzhou University, Zhengzhou, China

**Keywords:** workplace violence (WPV), hospital, health workers (HWs), anti-violence measures, occupational health

## Abstract

**Objective:**

Workplace violence (WPV) not only affects workers' physical and mental health but also increases turnover rates and social costs. There are no studies that have consistently followed the prevalence of WPV and the effectiveness of anti-violence measures in cross-sectional studies of the same hospital. The main aim of this study was to understand the prevalence of WPV among medical staff in a hospital in China and the effectiveness of implemented anti-violence measures.

**Methods:**

A cross-sectional survey, which was conducted by using a questionnaire developed by the International Labor Organization and the World Health Organization, was administered in a tertiary hospital in China in 2022 to investigate the occurrence of violence in the past year. Valid data were collected from 1,195 respondents *via* cell phone. WPV was compared between this survey and a baseline survey conducted at the same hospital in 2017 using the same questionnaire. The relationship between years and gender and occupation was explored using the Cochran–Armitage trend test. Descriptive statistics and chi-square tests were used to respond to the effects of the anti-violence interventions.

**Results:**

After the implementation of anti-violence measures, the prevalence of WPV decreased from 48.47 in 2017 to 33.95% in 2021. The recognition of safety measures increased from 80.16 in 2017 to 87.70% in 2021(*P* < 0.001), improvement of the work environment increased from 56.57 in 2017 to 65.10% in 2021(*P* < 0.001), restricted of public access increased from 34.36 in 2017 to 55.60% in 2021(*P* < 0.001), the patient protocols increased from 37.42 in 2017 to 38.40% in 2021, patient screening increased from 32.11 in 2017 to 41.90% in 2021(*P* < 0.001), and strict restrictions on workplace cash activities from 29.65% in 2017 to 36.00% in 2021(*P* < 0.05). The percentage of health workers who felt that anti-violence measures were not at all helpful to their current work decreased from 2017 to 2021.

**Conclusions:**

The prevalence of workplace violence has decreased, recognition of interventions has increased, and the proportion of interventions that are perceived by HWs as not helpful at all has decreased. This study demonstrates that the 12 anti-violence measures recommended by the International Labor Organization are applicable in China. Based on this, hospitals should focus on the improvement of the intervention effect of the following measures to further prevent violence.

## Introduction

Workplace violence (WPV) has become a global social problem, affecting health workers (HWs) at a higher rate than other occupations (1, 2). WPV affects the physical and mental health of HWs, and increases the turnover rates and economic burden on society (2–4). Previous studies evaluating workplace aggression have shown that 7–82.9% of HWs are targets of violence (5–11). In China, the prevalence of WPV among HWs ranges from 14.26–69.24% (12–15). However, there are still gaps in the mid-term and long-term descriptions of WPV in healthcare, especially with reference to the effectiveness of mid-term and long-term WPV interventions (16–19). These studies identified several influencing factors that can inform subsequent refinement of interventions to improve the safety of HWs.

WPV has been classified into four types based on the correlation between the perpetrator and the workplace. Type II WPV in hospitals refers to verbal or physical attacks on HWs by patients and visitors. In fact, hospital settings are more likely to trigger Type II WPV than other environments (11, 20, 21). The follow-up on anti-violence measures should elaborate on their lasting effects, as these are not addressed by short-term and or single surveys. Currently, in China, there is a shortage of medium- and long-term sustained follow-up surveys of the improvement measures.

There are different tools for the assessment of WPV among HWs. Current research instruments on WPV in HWs mostly use the self-report questionnaire, a self-administered questionnaire, a short-term assessment tool, the Crisis Monitor, the Brøset Violence Checklist (BVC), the Brief Psychiatric Rating Scale (BPRS), the Perception of Aggression Scale (POAS), and the Perception of Patient Initiated Aggression Tool (POPAS) (22–27). Currently, most of the questionnaires that are used within China are homemade, or only a part of the questionnaire is utilized (13, 28, 29), which indicates a lack of sufficient studies using internationally recognized survey instruments.

Many countries have developed prevention and control guidelines and technical tools to assess WPV. E.g., The Italian Ministry of Health issued recommendations in 2007 for the prevention and management of violence among healthcare workers (30). Australia has developed a risk assessment system for patients, which helps to identify patients as “at risk” when they exhibit one or more of the “at risk” signs in the assessment system. Also, this system involves making a special note in the medical record to alert other patients, departments, and staff and promptly notify the relevant security department (31). The U.S. Occupational Safety and Health Administration (OSHA) issued the “Guidelines for preventing workplace violence for health care social service workers” in 2004 (32), which consists of several specific provisions aimed at creating a safe healthcare environment. Besides HWs' own prevention skills, these measures include the establishment of a comprehensive self-prevention system at the hospital level, such as metal detectors, alarm buttons, 24-h monitoring systems, emergency evacuation, and access control systems. The International Council of Nurses, Public Services International, the World Health Organization (WHO), and the International Labor Organization jointly developed and published “Framework guidelines for addressing workplace violence in the health sector: the training manual” (33). Interim guidance issued by the International Labor Organization and WHO during Coronavirus Disease 2019 (COVID-19) highlights the need to strengthen the prevention and control of violence, harassment, and discrimination experienced by HWs.

About 25% of WPV incidents have been reported to occur in the health sector (2). The International Labor Office (ILO), the International Council of Nurses (ICN), the WHO, and Public Services International (PSI) co-sponsored a program that conducted case studies in Brazil, Bulgaria, Lebanon, Portugal, South Africa, Australia, Thailand in 2000 aiming to prevent and eliminate violence in the health sector. The 12 measures provided in the questionnaire “Workplace violence in the health sector country case studies research instruments: survey questionnaire” (hereafter referred to as the “Questionnaire”), which were jointly developed by these four organizations, are widely recognized as comprehensive anti-violence measures (34).

The present study was based on the ILO/PSI/WHO/ICN definition of WPV (33). This retrospective cross-sectional survey was conducted at the third People's Hospital of Zhengzhou (Zhengzhou, Henan, China) in 2022 using an international survey instrument to investigate the WPV, which occurred from May 31, 2021 to May 31, 2022 and for an accurate description of our survey based on this article on the 2021 survey), and after the 2018 survey (to investigate the occurrence of violence from May 31, 2017–2018. For an accurate description of our survey, we referred to the 2017 survey or the baseline survey), we took steps to address the occurrence of WPV according to the guidelines and compared changes from the 2018 survey results based on the corresponding measures (35). To assess the current prevalence of WPV amongst the HWs at a tertiary care hospital in China in comparison to a previous prevalence of WPV in the same institution. Moreover, the study aimed to identify the effectiveness of the implemented anti-violence measures in our hospital.

## Methods

### Design

The third People's Hospital of Zhengzhou is the pilot hospital of HealthWISE application which organized by Min Zhang's team in Peking Union Medical College (PUMC). As part of the pilot study, the cross-sectional survey was conducted in 2018, 2019 and 2021 with the same questionnaire and methodology, respectively, the methodology and results of those surveys were published previously elsewhere (33–35).

Based on previous results and methodology, a cross-sectional survey was focused on twelve anti-violence measures by distributing an online questionnaire. Our members of the research team visited the hospital for the survey and over 7 days in June 2022.

In June 2018, a retrospective cross-sectional survey was conducted in the same hospital using the same survey questionnaire and the same survey population (35), which in the present study was defined as a baseline survey.

The overall study leading by Min Zhang on nationwide occupational health protection for health workers in 2022 was approved by PUMC ethics committee (number CAMS&PUMC-IEC-2022-044). The study has been reviewed and approved by the Third People's Hospital of Zhengzhou ethics committee (approval number 2022-04-019-K01).

### Participants

This survey was conducted at the third People's Hospital of Zhengzhou (Central China tertiary general hospital that provides healthcare services across regions, provinces, cities, and nationwide, hereinafter referred to as “the hospital”) with 1,100 beds and 2,030 employees. The target population included all hospital healthcare workers, i.e., doctors, nurses, medical technicians, and administrative staff. We included clinical managers in our study as part of our administrative staff because they not only have extensive contact with HWs, but also play an important role in the development of anti-violence policies and are implementers and beneficiaries of anti-violence measures. The inclusion criteria were as follows: (1) professionally certified HWs; (2) voluntary participation in the survey with informed consent; and (3) employed by the hospital as a regular employee for >1 year. Exclusion criteria were the following: (1) those failing to answer the questionnaire in the opening hours; (2) those exceeding the time limit for the questionnaire.

### Questionnaire

The original language of the questionnaire was English (34). The pretest and retest reliability and validity were assessed by Prof. Min Zhang's team in Beijing and Shenzhen, with Cronbach's alpha (α) coefficient 0.83 (35). And we have obtained the authorization letter of the questionnaire from the team. Cronbach's α for a summary score of 0.70~0.80 is considered satisfactory for a reliable comparison between groups, and > 0.90 is required for the clinical usefulness of the instrument (36). In this study, the validity factor of the questionnaire was 0.854. This questionnaire included: (1) participants' demographic information (department, title, education level, age, salary, and gender); (2) experience of physical violence and post-event intervention in the past 12 months; (3) psychological violence in the past 12 months (including verbal abuse, bullying/mobbing, racial and sexual harassment), and post-event intervention experiences in the past 12 months; (4) hospital violence prevention measures and opinions on the causes of violence consisting of three open-ended questions.

### Data collection

In June 2022, members of the research team visited the relevant departments, and for more than 7 days, they kept inviting HWs on duty to fill out an online questionnaire using their cell phones. The online questionnaire included a consent statement on the first page regarding the anonymity, confidentiality, and voluntary participation. The Chinese version of the questionnaire is available online (https://www.wjx.cn/vm/emTu2I4.aspx). An online version of our questionnaire was distributed through WeChat, which allowed HWs to access and participate in our survey by scanning a specific quick response code from their own phone or tablet. WeChat is the most widely used mobile social application in China, with over 1.2 billion active users in 2022 (37). HWs participated in our survey as WeChat users and in different WeChat groupings according to different department names. Therefore, this was an effective way to implement surveys and communication.

The target sample size was set to 1600 HWs for the whole hospital in order to include every department of medical workers in our research scope. At the same time, this survey method was consistent with the baseline survey, which is more conducive to comparing the research effect of our anti-violence measures. A total of 1,195 individuals fulfilled the inclusion criteria. The data management platform showed that 917 questionnaires were completed by respondents who met the inclusion criteria, 913 of which were valid (total response rate 76.74%; total valid response rate 76.40%).

### Quality control

Before the survey, the team members explained the concept, purpose, and importance of the survey. After collecting the questionnaire from the online platform, two team members validated the data and removed or corrected the logical errors with mutual consent.

### Statistics

The database was coded, and SPSS V.26.0 (SPSS Inc., Chicago, IL, USA) and Excel were used to perform data analysis.

The independent variable is the year, and the dependent variables are gender and occupation. Categorical variables were statistically described by frequency (percentage). The relationship between different years and gender and occupation was explored using the Cochran–Armitage trend test.

First, the gender and occupation of the participants in both surveys were examined using the Cochran-Armitage (CA) trend test.

Second, workplace violence responses were described and evaluated. The descriptive statistics of the total prevalence of violence, the prevalence of various types of violence, responses, medical staff approval of anti-violence measures, and the extent to which medical staff found anti-violence measures helpful were compared between the two surveys using Excel and SPSS V.26.0. *P* < 0.05 indicated statistical significance. We evaluated the effectiveness of the violence measures by comparing the results of the baseline survey with those of the current survey, it included prevalence of workplace violence, prevalence of various types of workplace violence, recognition of anti-violence measures, extent to which health care workers find anti-violence measures helpful. The trend chi-square test was used to compare changes in attitude medical workers had toward various measures between this survey and the annual baseline survey.

## Results

### Changes in the prevalence of WPV

**Figure 1:**
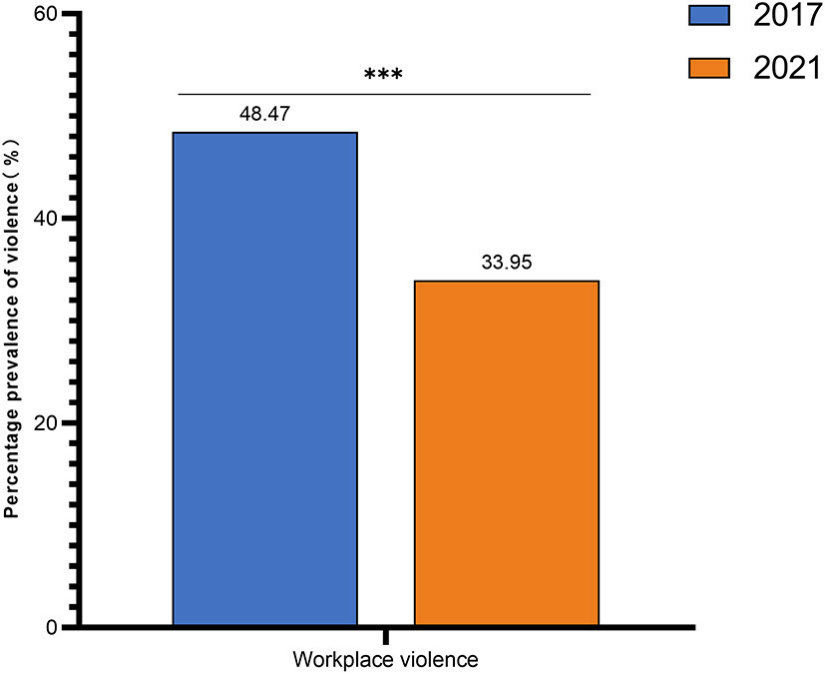
Statistical chart of the change in the total prevalence of violence. Both 2017 and 2021 in the figure refer to the year. The prevalence of WPV in hospitals decreased from 48.47 in 2017 to 33.95% in 2021. ^***^indicates that *P* < 0.001. Total workplace violence prevalence, Pearson's Chi square = 40.974, *P* < 0.001.

**Figure 2:**
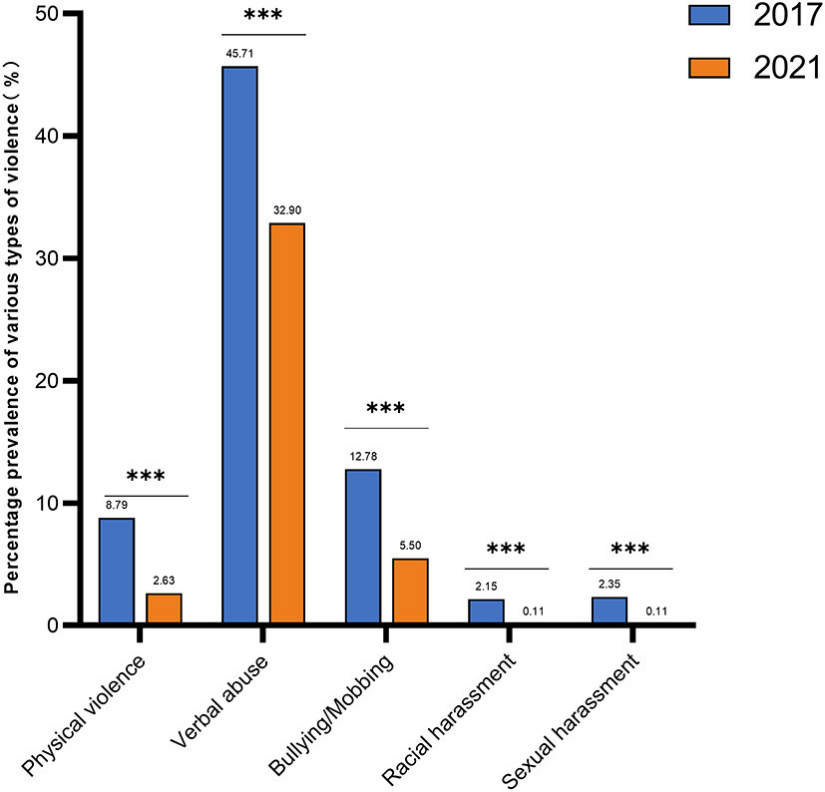
Graph of changes in the prevalence of various types of violence among HWs. Both 2017 and 2021 in the figure refer to the year. From 2017 to 2021, all types of violence decreased. The prevalence of verbal abuse has been the highest. Also, the statistical analysis of the two surveys showed the largest decrease in verbal abuse. ***indicates that *P* < 0.001. Physical violence, Pearson's Chi square = 32.775, *P* < 0.001; Verbal abuse; Pearson's Chi square = 32.610, *P* < 0.001; Bullying/Mobbing, Pearson's Chi square = 30.002, *P* < 0.001; Racial harassment, Pearson's Chi square = 17.051, *P* < 0.001; Sexual harassment, Pearson's Chi square = 18.945, *P* < 0.001.

For gender, using the trend test, i.e. Cochran-Armitage (CA) trend test, the *p*-value of the trend test is < 0.05, indicating that the change in gender over time is not statistically significant for the trend(χ*2*_*trend*_ = 3.757, *P* = 0.053)For occupation, using the trend test, i.e., Cochran-Armitage (CA) trend test, the *p*-value of the trend test is < 0.05(χ*2*_*trend*_ = 193.481, *P* < 0.001), indicating that the change in occupation has a trend statistical significance over time, with the proportion of nurses increasing year by year and the number of doctors and technical support and administrative staff decreasing year by year (Table 1). The total prevalence of violence decreased from 48.47 in 2017 to 33.95% in 2021 (Figure 1). Physical violence decreased from 8.79 to 2.63%. Verbal abuse decreased from 45.71 to 32.90%, while bullying/ mobbing decreased from 12.78 to 5.50%, racial harassment decreased from 2.15 to 0.11%, and sexual harassment decreased from 2.35 to 0.11% (Figure 2).

**Table 1:** Overview and description of HWs included by gender and occupation.

		**2017 (*****n*** = **978)**		**2021 (*****n*** = **913)**		
**Characteristic**		***n***	**%**	***n***	**%**	***χ2***	***p*-value for trend**
Gender						3.757	0.053
	Male	177	18.10	135	14.79		
	Female	801	81.90	778	85.21		
Occupation						193.481	< 0.001
	Technical support and administrative staff	171	17.48	129	14.13		
	Doctors	255	26.07	227	24.86		
	Nurse	552	56.44	557	61.01		

### Changes in recognition of anti-violence measures in the workplace

**Figure 3:**
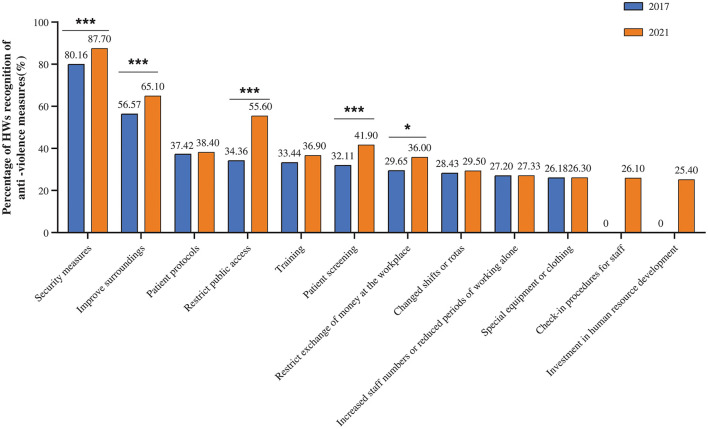
Statistical chart of the percentage of recognition of anti-violence measures in the workplace. Both 2017 and 2021 in the figure refer to the year. In both surveys, security measures ranked first in terms of recognition. The recognition of restricting public access showed the largest increase. ***indicates that *P* < 0.001. *indicates that *P* < 0.05. Security measures, Pearson's Chi square = 20.434, *P* < 0.001; Improve surroundings, Pearson's Chi square = 13.682, *P* < 0.001; Restrict public access, Pearson's Chi square = 86.568, *P* < 0.001; Patient screening, Pearson's Chi square = 19.658, *P* < 0.001; Patient protocols, Pearson's Chi square = 0.209, *P* = 0.647; Restrict exchange of money at the workplace, Pearson's Chi square = 8.736, *P* < 0.05; Increased staff numbers or reduced periods of working alone, Pearson's Chi square = 0.001, *P* = 0.971; Special equipment or clothing, Pearson's Chi square = 0.003, *P* = 0.956; Changed shifts or rotas, Pearson's Chi square = 0.247, *P* = 0.619; Training, Pearson's Chi square = 2.504, *P* = 0.114.

The comparison of the results of this and the baseline surveys revealed that the percentage of approval of public access control is increased maximally from 34.36 in 2017 to 55.60% in 2021, followed by the percentage of approval of patient screening from 32.11 to 41.90%, the percentage of approval of improvement of surrounding from 55.67 to 65.10%, the percentage of recognition for safety measures from 80.16 to 87.70%, the percentage of recognition for restricting the exchange of money in the workplace from 29.65 to 36.00%, the percentage of recognition for patient protocol from 37.42 to 38.40%. The percentage of recognition for training percentage of recognition increased to 36.90% in 2021 from 33.44% in 2017, and the recognition of changing shifts or rotas increased to 29.50% in 2021 from 28.43% in 2017. Increasing staff numbers or reducing the periods of working alone, special equipment or clothing showed a flat percentage increase in recognition at 0.13 and 0.12%, respectively. Among these, the check-in procedures for staff and investment in human resource development are new measures added after the baseline survey, with percent approval of 26.10 and 25.4%, respectively, in this survey (Figure 3).

### Changes in the extent to which anti-violence measures are perceived by HWs as helpful to existing efforts

**Figure 4:**
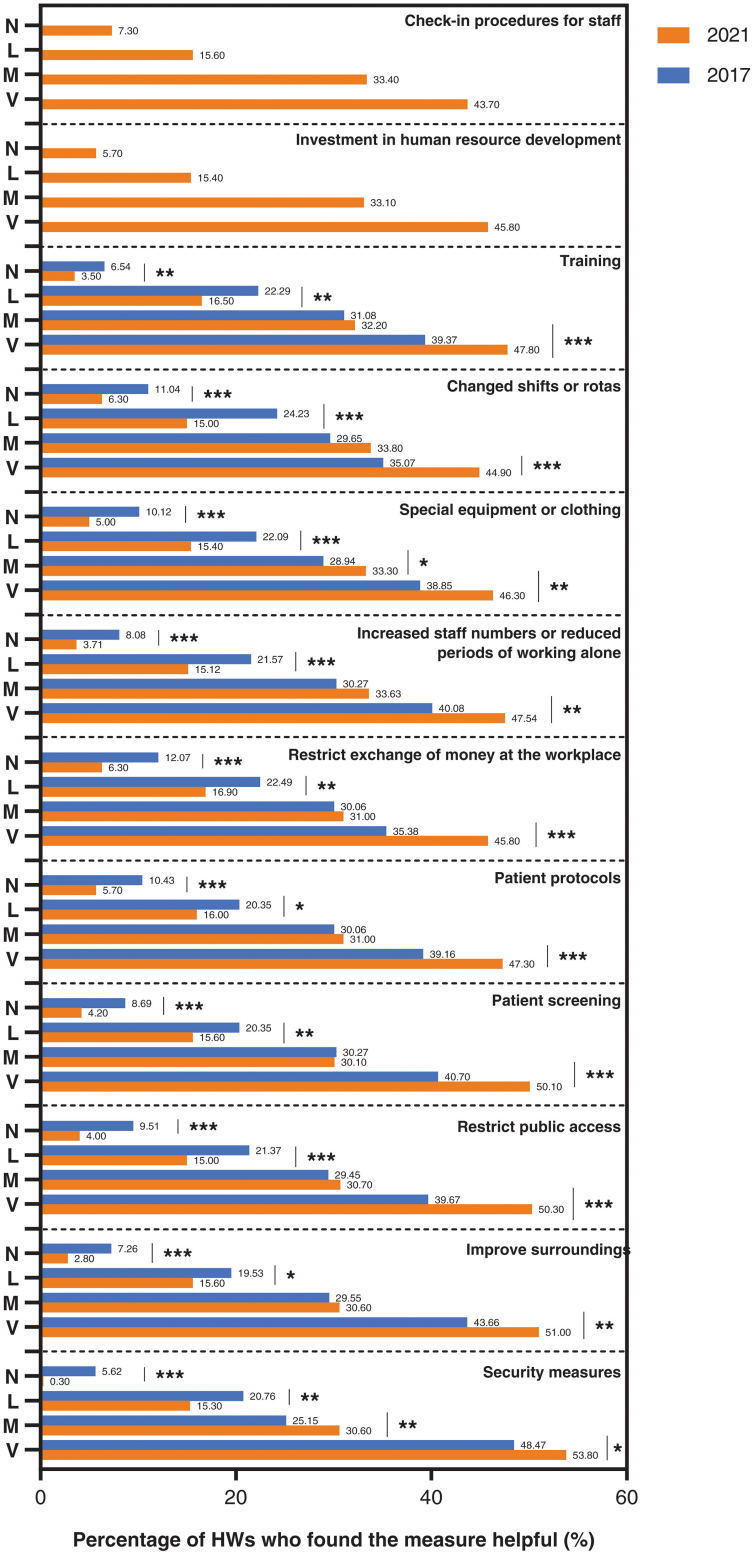
The extent to which healthcare workers find various types of measures useful in their existing work. Both 2017 and 2021 in the figure refer to the year. V, M, L, and N stand for very, moderate, little, and not at all, indicating very helpful, moderate, slightly helpful, and not helpful, respectively. ***indicates that *P* < 0.001. **indicates that *P* < 0.01. *indicates that *P* < 0.05. Security measures(not at all): Pearson's Chi square = 44.531,*P* < 0.001. Improve surroundings (not at all): Pearson's Chi square = 18.887, *P* < 0.001Restrict public access (not at all): Pearson's Chi square = 21.961, *P* < 0.001. Patient screening (not at all), Pearson's Chi square = 15.052, *P* < 0.001; Patient protocols (not at all), Pearson's Chi square = 14.146, *P* < 0.001; Restrict exchange of money at the workplace (not at all), Pearson's Chi square = 18.256, *P* < 0.001; Increased staff numbers or reduced periods of working alone (not at all), Pearson's Chi square = 15.930, *P* < 0.001; Special equipment or clothing (not at all), Pearson's Chi square = 18.106, *P* < 0.001; Changed shifts or rotas (not at all), Pearson's Chi square = 13.659, *P* < 0.001; Training (not at all), Pearson's Chi square = 9.050, *P* < 0.01.

A comparison of this survey with the baseline survey revealed that the largest percentage decrease in measures was valuable for restricting the exchange of money (decreased by 5.77%), followed by restricting public access at the workplace (decreased by 5.51%), security measures (decreased by 5.32%). The special equipment or clothing (decreased by 5.12%), changing shifts or rotas (decreased by 4.74%), patient protocols (decreased by 4.73%), patient screening (decreased by 4.49%), surrounding improvements (decreased by 4.46%), increased staff numbers or reduced periods of working alone (decreased by 4.37%), and the smallest decrease in training among the above ten items (decreased by 3.04%). Investment in human resource development and check-in procedures for staff are new measures added after the baseline survey, and the proportion of individuals considered not helpful at all in this survey is 5.7 and 7.3%, respectively. However, in both surveys, the percentage of security measures was consistently the minimum. With the exception of two measures, the investment in human resource development and the check-in procedures for staff, the percentage of the remaining ten measures that were perceived by HWs as not at all helpful decreased, and all were statistically different (Figure 4).

## Discussion

Between 2000 and 2002, ILO/ICN/WHO/PSI studied all the healthcare providers in 7 countries (Brazil, Bulgaria, Lebanon, Portugal, Thailand, South Africa, and Australia) using the same questionnaire as was used in the present study, finding that the majority of the healthcare workers had experienced at least one prevalence of physical or psychological violence, amounting to following rates: 75.8% in Bulgaria, 67.2% in Australia, 61% in South Africa, 60% in a medical center and 37% in a hospital in Portugal, 54% in Thailand, and 46.7% in Brazil (2). After the baseline survey, the 12 violence risk prevention measures of the questionnaire served as a guide for the implementation of appropriate violence risk prevention measures in our hospital, resulting in an obvious decrease in the prevalence of violence, going from 48.47 in 2017 to 33.95% in 2021 (33, 38).

### Security measures

Security is one of the major anti-violence measures, which consistently had the highest HWs' endorsement (80.16 in 2017 and 87.7% in 2021) and was consistently considered the most helpful anti-violence measure (48.47 in 2017 and 53.80% in 2021). We also improved the WPV early warning system by installing security cameras and providing the HWs with walkie-talkies to allow timely contact with security staff in dangerous situations, improved the departmental WPV reporting process and added a WPV self-reporting checklist, and revised the hospital's WPV risk contingency plan. Next, the number of guards (55.6% increase from the baseline survey), guard rooms (an additional new room), and patrols were increased, the security guards were provided electric batons, and the hospital infrastructure was strengthened. A cross-sectional study on the security measures for WPV in hospitals (39). However, the necessity of metal detectors for hospital admission and their effectiveness still remains controversial. Since the hospital is a public place, it is difficult to control the source of metals. Patients can also get hold of such objects from the hospital or bypass the security system by entering the hospital premises in an ambulance. Thus, how to control the source of forbidden items to enhance security (for example, some patients or visitors themselves carry items that could be used as weapons at any time, such as lighters, sharp jewelry, etc. Moreover, emergency patients coming in through the ambulance corridor may also carry some dangerous items) which should be further explored.

### Restricted public access

Among our measures implemented to reduce the prevalence of WPV, the increase in the percentage of acceptance of public access control ranked first (34.36 in 2017 and 55.60% in 2021). As visitors are one of the major sources of violence in healthcare settings (40), we adopted the following measures to limit public access. Previous studies also confirmed the validity of the present study's results (41). Similarly, controlling public access was shown to reduce the prevalence of WPV in an intervention study in China (42). We also noted that this measure might increase the risk of WPV among security personnel while reducing the prevalence of WPV in HWs. Therefore, how to reduce the overall prevalence of violence in hospitals requires further comprehensive investigation.

### Increased staff number or reduced periods of working alone

Increasing the staff or reducing independent work hours led to a modest increase in approval (0.13%) of the measures across two surveys. With a rate of 4.37%, the percentage decrease for this measure was considered the least helpful. The presence of coworkers can diminish a potential assault threat (43). The same recommendation was made in a proposal for an action plan to organize emergency services in Norwegian primary healthcare (44). Therefore, it is worrying that most HWs work alone in some cases. Since the baseline survey, the number of in-ward caregivers has increased by 45% based on the third-party professional in-hospital companions contracted by the hospital, which has improved the quality of in-hospital care while reducing violence and lowering economic costs. Concurrently, the hospital increased the recruitment of HWs and implemented an in-hospital talent pool strategy, assigned medical interns to departments with high rates of WPV, and had at least two individuals working together during times of high violence, thus effectively reducing the prevalence of WPV. Also, the interventions to increase staff or reduce independent work time had a positive effect.

### Check-in procedures for staff

With the launch of the “Ubiquitous Medical” model (45), we have developed a series of systems and operational procedures for home care services, including timely positioning and clocking in according to the operational procedures as a critical measure to ensure the safety of home care workers. The importance of this measure was also recognized by another study on risk control strategies for home care in China (46) and was further confirmed by the present survey. However, the current assurance measures and evaluation indicators for operational and provider safety need further improvement. Thus, we suggested developing systematic and scientific evaluation indexes and assessment tools to evaluate the quality of nurses' home care services at three levels, i.e., structure, process, and outcome, which could help further exploit the role of intelligent telemedicine devices in safety assurance.

### Patient screening

Unlike measures such as patient violence medical records in other countries (47), our understanding of the occurrence of violence in the department was mainly based on the information shared during the morning meeting of the department or mentioned in the handover process. For example, patients and family members with a history of violence in medical settings, the occurrence of violence in the department on the previous day, patients or family members who were emotionally unstable during treatment, and the work logs of doctors and nurses. Following the end of the baseline survey, we added a new departmental violence handover register, which includes time, place, type of violence, the way the violence occurred, departmental treatment, and treatment outcomes. Currently, this measure is not considered very useful, as in our survey from 2017, it was 8.69% declining to 4.20% in 2021.

### Improve surroundings

In the present study, working surroundings consisted of light wood furniture without sharp corners that were attached to the floor in parlors or places prone to violence. We also replaced the damaged lamps to maintain bright indoor and outdoor lighting. For some departments with a high prevalence of WPV, HWs should administer dedicated disposal or operating rooms to manage patients. A third-party cleaning company is contracted in the hospital to take charge of the wards, outpatient clinics, and dining restaurants in separate areas to improve the neatness and comfort of the hospital environment. Since the building structure of our old hospital area could not be changed, we set up screen partitions, while in the new hospital area, we set up two consultation rooms before the opening of the wings. The HWs' approval increased by 8.53% for these measures. A German study showed that improvements in the work environment could reduce the prevalence of WPV (48). Thus, we supposed that even if the structure of the building cannot be changed, it is still possible to decrease the WPV rate. Since the measures related to counseling rooms are difficult to implement without changes to the building structure, the above measures must be included in the initial planning phase of violence interventions. Barriers or glass partitions in the reception area are common and can usually be implemented without major structural modifications.

### Restrict exchange of money at the workplace

According to the FIS 2022 Global Payments Report data, digital wallets, such as Alipay and WeChat Pay, are the leading payment options for Chinese consumers, accounting for about 83% of total e-commerce transactions in 2021 (49). With the merging of the health insurance cost settlement platform with the e-Health service platform and the increase in health insurance reimbursement rates, the process of paperless in-hospital payments was further advanced. However, unlike the pay-as-you-go health insurance system outside of China (50), payment before seeing a doctor is still a predominant modality within the country. Currently, payment for medical expenses in Europe and USA is mainly achieved by credit card, and the use of digital wallets is still relatively low (51). However, there is increasing use of third-party payments in Chinese hospitals. Although we have a slightly different approach to restricting the use of cash, the efficiency of this approach has been well-proven.

### Training

We encouraged our staff to attend HealthWise courses and also provide training for HWs (52). In addition, a social media group on WPV prevention and control has been set up, occupational safety information is dispensed, and a quiz on related knowledge was set up (https://weixin.qq.com/g/AwYAAIOgcnYzDwtv0JB1JauhzBOhojD9FzN6sqVLkKcmtA3ib0ERPDtmJEvglITa). A virtual simulation of the intelligent classroom in the workplace for emergency violence response process simulation training was also conducted. The public security departments of the streets and lawyers from local law firms were invited to impart judicial knowledge land to train the hospital employees on laws and regulations in health care, respectively. The training methods for WPV are mainly based on face-to-face or online courses, which are consistent with the measures taken in this study (16, 19, 53). The effectiveness of the training has been proven by the satisfactory intervention results observed in several studies (13). The 5-year interval between surveys in this study provides evidence of the effectiveness of adherence training; however, there is a lack of long-term research on this measure, which needs to be further explored in future studies.

### Patient protocols

Upon admission, patients or their families are required to sign an informed consent form for some expensive medications and medical services that are not covered by medical insurance. The informed consent form is signed after admission to the hospital, and the nurse on duty is informed when leaving the room during the hospital stay, especially for some patients who need to reduce their activities. This is not the same as the patient privacy confidentiality agreement referred to in most U.S. state patient agreements (54). China's Nine Guidelines for the Integrity of Health Care Facility Staff clearly state that the principle of confidentiality should be observed and patient information should not be disclosed (55). This is both a legal and ethical requirement for HWs.

### Special equipment or clothing

Hospitals are equipped with batons, body shields, walkie-talkies for security guards, and walkie-talkies for departments with a high prevalence of violence (such as emergency departments). Several studies have confirmed the necessity and effectiveness of metal detectors (56–58). However, this study did not adopt metal detectors due to the uncertainty of the security source and to avoid unnecessary tension and panic.

### Changed shifts or rotas

Since the baseline survey, our hospital has initiated a system to update the schedules of doctors and nurses every 2–4 weeks to avoid repeatedly working with the same individual at a time with a high prevalence of violence in a short period. This helps reduce the prevalence of violence and burnout among healthcare workers, thereby enhancing the safety of healthcare workers and patients (59).

### Investment in human resource development

The hospital's pay and performance reform, which added quality control of the staff and linked the service quality to pay and performance, was well received within the hospital, with only 5.7% of the participants reporting it as not helpful in 2021 in the present survey. These results were confirmed in a prospective study on the cross-sectional association between social capital and low rates of WPV (60).

Currently, the main international measures are safety measures, environmental improvements, training, and organizational interventions, of which training and organizational interventions have been proven to be safe and effective. This study's measures, work environment improvement, and restricted public access reached >50% success. Therefore, the next step is to further improve the effectiveness of implementing anti-violence measures.

The current international concentration on organizational intervention research, which emphasizes how the intervention process is conducted and implemented to ensure quality, would provide a new perspective for our study. In addition, the training measure is the most studied worldwide. Although this measure made a difference in both surveys, the observed improvement was small, which is consistent with the results of a previous meta-study on education and training (13). Therefore, improving the effectiveness of medium- and long-term education and training also provides new research directions on how to ensure the effectiveness of training.

Both surveys were conducted in the same hospital to reflect the continuity and effectiveness of the measures on the occurrence of violence in the past 12 months: a baseline survey was conducted from May 31, 2017 to May 31, 2018, and a second survey was conducted from May 31, 2021 to May 31, 2022, with the two datasets independent of each other. For both surveys, we calculated the total violence prevalence according to the number of people, and also each type of violence was calculated according to the number of people, which was based on the possibility of a single person experiencing multiple types of violence.

In the national reports of seven countries, safety measures were introduced first, followed by improvements in the working environment (2). In our 2021 study, safety measures had an 87.7% approval rating, which was the highest compared to other countries (2). In Brazil, when asked, “To what extent do you think these measures contribute to your work environment?,” the respondents ranked security measures as very helpful, ranking third in that study but first in our study (2). The recognition of the improvement in the surroundings has been more obvious than in Bulgaria (48.8%) but lower than in Thailand (77%) (2). Of these, the recognition of public access control has increased the most in both our surveys (21.24% increase) and was higher in this survey (55.6%) than in Bulgaria (27.3%), but a higher percentage of Bulgarian survey participants (58.9%) than in ours (2021, 50.30%) believe that limiting public access is the most effective measure (2). In the current study, compared to the baseline survey, we have a breakthrough in terms of investment in human resource development and increased punch card systems in the workflow, which is closely related to the continuous improvement of the anti-violence measures. However, direct comparisons across countries require careful consideration, especially regarding the intrinsic validity and reliability of each questionnaire. The comparison of the two surveys revealed an increase in the percentage of recognition of our measures among survey participants who found each measure very useful. Due to the diversity of health institutions, and economic and sociocultural contexts across survey years, the validity of the measures could be cross-referenced across countries. With the advancement in the laws and rules of civilization in society, the increase in positive image promotion videos of HWs has also increased the public awareness and tolerance of the work content and environment of HWs, which in turn has contributed to the implementation process of anti-violence measures in the workplace.

Regarding the effectiveness of the implementation of the above 12 anti-violence measures, the significant reductions in total workplace violence, physical violence, verbal abuse, and bullying/mobbing, racial harassment, sexual harassment, the increase in recognition of countermeasures, and the decrease in the percentage of measures considered not at all helpful in the level of help after the baseline survey suggest that these measures are effective but still need further improvement. For example, the prevalence of verbal violence continues to have the highest of all violence rates, and increasing staff numbers or reducing the periods of working alone, special equipment, or clothing showed a flat percentage increase in recognition. Based on the results of the baseline survey, we formulated the Regulations on the Management of Violence Prevention Measures according to the above guidelines and guidelines, which mainly included the three-level reporting process of workplace violence risk (individual, department and medical Administration and management department), relevant countermeasures and implementation rules. Through our research, the effectiveness of our measures has been proven to some extent. Based on this, our hospital will improve our violence risk management, emergency response mechanism, violence prevention, and response measures so as to better improve the effectiveness of our anti-violence measures. The hospital's measures to prevent and control workplace violence have had certain effects, but there are still areas to be improved from the comprehensive measures proposed after the previous survey. Based on the results of this survey, the following improvement measures are suggested:

Hospital should further improve the risk assessment system, and to direct contact with the patient/family/strangers in risk assessment, risk identification of special situation (such as shift, work alone, etc.), in a reasonable analysis on the basis of the characteristics of the environment, according to the level of risk classification management measures, make a targeted prevention programs, change passive response to active prevention and control, To prevent violence. Workplace policies and plans to combat workplace violence should be developed, with the necessary monitoring mechanisms and scope for sanctions; Improve reporting and recording systems and encourage the reporting of all incidents involving physical and psychological violence, as well as minor and potential incidents that do not result in actual harm (recommendations can also be made to reduce the risk of violence or improve working conditions); Reports of such incidents are regularly reviewed as indicators of improved workplace safety measures. The union should regularly assess the risk of workplace violence with hospital leaders and make improvements based on the results.

## Limitations and strengths

This study used a retrospective approach to investigate the occurrence of violence over 12 months with a recall bias. Hence, we considered the pre-survey training on the knowledge and re-explanation at the time of the survey, and allowed the HWs to review their work logs for records of violence. As a cross-sectional study, it was not possible to analyze the causal relationships between variables.

We implemented our online questionnaire through a scannable quick-response code developed by social media, which is available and is currently used in several medical-related survey studies (33, 34, 61–64). The survey used an internationally accepted questionnaire to develop corresponding comprehensive measures using internationally accepted anti-violence measures as a guide, and the increase in recognition of these measures and the decrease in the percentage of measures that were considered unhelpful both enhance international comparability and support the adoption of similar or further comprehensive interventions or policies in Chinese hospitals. Moreover, at present, our team is among the few in China that have used an internationally recognized tool to validate the effectiveness of workplace violence measures.

## Conclusion

As in most countries around the world, there is a constant risk of violence in Chinese hospitals, which can affect the physical and mental health of medical workers, their productivity, and possibly increase their sense of job burnout. This study demonstrated that the 12 violence risk prevention measures recommended by the ILO questionnaire are effective in Chinese tertiary care hospitals, especially in terms of security measures, improvement in the work environment, public access control, and patient screening. However, we still need more measures to improve the effectiveness of our interventions. Although these measures can be replicated, their implementation needs to be integrated with the country's cultural, economic, and social environment.

## Data availability statement

The raw data supporting the conclusions of this article will be made available by the authors, without undue reservation.

## Ethics statement

The studies involving human participants were reviewed and approved by the PUMC and the Third People's Hospital of Zhengzhou of the Ethics Committee. The patients/participants provided their written informed consent to participate in this study.

## Author contributions

MZ designed and organized the pilot study in Third People's Hospital of Zhengzhou since 2017. MZ, RL, and NC conducted the survey in 2018. MZ, RL, NC, YLiu, and YHua conducted the survey in 2019. RL, MZ, YW, QL, and YHu contributed to design of the study in 2022. YHu organized the database and wrote the first draft of the manuscript. YHu and PS performed the statistical analysis. JS and YLi wrote sections of the manuscript. MZ, QL, YLiu, YHua, and NC made the final revision of the manuscript. All authors contributed to manuscript revision, read, and approved the submitted.
