# Supplementary material for: Anti-violence measures developed by ILO and WHO: Analysis of the prevalence of workplace violence and the effects of implementation in a general hospital in China
Source: Front Public Health. 2022 Dec 14;10:1049832. doi: 10.3389/fpubh.2022.1049832 (PMC9794770; doi:10.3389/fpubh.2022.1049832)
Supplement: Supplementary file 1 [file Data_Sheet_1.PDF]

frontiers

Supplementary Material and Manuscript ID: 1049832

Table 1 Total prevalence of workplace violence, prevalence of various types of workplace violence, and recognition of anti-violence measures

		2017(*n*=978)		2021(*n*=913)			
**Characteristic**		**n**	**%**	***n***	**%**	***χ2***	***P value***
Type						42.679	＜0.001
	Workplace violence	474	48.47	310	33.95		
	Physical violence	86	8.79	24	2.63		
	Verbal abuse	447	45.71	300	32.90		
	Bullying/Mobbing	125	12.78	50	5.50		
	Racial harassment	21	2.15	1	0.11		
	Sexual harassment	23	2.35	1	0.11		
HWs recognition of anti -violence measures						451.698	＜0.001
	Security measures	784	80.16	801	87.70		
	Improve surroundings	555	56.57	594	65.10		
	Patient protocols	366	37.42	351	38.40		
	Restrict public access	336	34.36	508	55.60		
	Training	327	33.44	337	36.90		
	Patient screening	314	32.11	383	41.90		

Continued Table 1

Characteristic		2017(*n*=978)		2021(*n*=913)			
		n	**%**	***n***	**%**	***χ2***	***P value***
HWs recognition of anti -violence measures							
	Restrict exchange of money at the workplace	290	29.65	329	36.00		
	Changed shifts or rotas	278	28.43	269	29.50		
	Increased staff numbers or reduced periods of working alone	266	27.20	250	27.33		
	Special equipment or clothing	256	26.18	240	26.30		
	Check-in procedures for staff			238	26.10		
	Investment in human resource development			232	25.40		

Table2 The extent to which HWs found anti-violence measures helpful

Characteristic			2017(*n*=978)		2021 (*n*=913)			
			n	**%**	***n***	**%**	***χ2***	***P value***
Extent of help								
	Security measures						58.401	<0.001
		very	474	48.47	491	53.80		
		moderate	246	25.15	279	30.60		
		little	203	20.76	140	15.30		
		not at all	55	5.62	3	0.30		
	Improve surroundings						27.764	<0.001
		very	427	43.66	466	51.00		
		moderate	289	29.55	279	30.60		
		little	191	19.53	142	15.60		
		not at all	71	7.26	26	2.80		
	Restrict public access						42.987	<0.001
		very	388	39.67	459	50.30		
		moderate	288	29.45	280	30.70		
		little	209	21.37	137	15.00		
		not at all	93	9.51	37	4.00		

Continued Table 2

			**2017(*n*=978)**		**2021 (*n*=913)**			
**Characteristic**			***n***	**%**	***n***	**%**	***χ2***	***P value***
Extent of help								
	Patient screening						29.236	<0.001
		very	398	40.70	457	50.10		
		moderate	296	30.27	275	30.10		
		little	199	20.35	142	15.60		
		not at all	85	8.69	39	4.20		
	Patient protocols						25.327	<0.001
		very	383	39.16	432	47.30		
		moderate	294	30.06	283	31.00		
		little	199	20.35	146	16.00		
		not at all	102	10.43	52	5.70		
	Restrict exchange of money at the workplace						36.906	<0.001
		very	346	35.38	418	45.80		
		moderate	294	30.06	283	31.00		
		little	220	22.49	154	16.90		

Continued Table 2

Characteristic			2017(*n*=978)		2021 (*n*=913)			
			n	**%**	***n***	**%**	***χ2***	***P value***
Extent of help								
	Restrict exchange of money at the workplace							
		not at all	118	12.07	58	6.30		
	Increased staff numbers or reduced periods of working alone						33.331	<0.001
		very	392	40.08	434	47.54		
		moderate	296	30.27	307	33.63		
		little	211	21.57	138	15.12		
		not at all	79	8.08	34	3.71		
	Special equipment or clothing						36.869	<0.001
		very	380	38.85	423	46.30		
		moderate	283	28.94	304	33.30		
		little	216	22.09	141	15.40		

Continued Table 2

			2017(*n*=978)		2021(*n*=913)			
**Characteristic**			***n***	**%**	***n***	**%**	***χ2***	***P value***
Extent of help								
	Special equipment or clothing							
		not at all	99	10.12	45	5.00		
	Changed shifts or rotas						46.887	<0.001
		very	343	35.07	410	44.90		
		moderate	290	29.65	309	33.80		
		little	237	24.23	137	15.00		
		not at all	108	11.04	57	6.30		
	Training						24.272	<0.001
		very	385	39.37	436	47.80		
		moderate	311	31.08	294	32.20		
		little	218	22.29	151	16.50		
		not at all	64	6.54	32	3.50		

Continued Table 2

Characteristic			2017(*n*=978)		2021 (*n*=913)			
			n	**%**	***n***	**%**	***χ2***	***P value***
Extent of help								
	Investment in human resource development							
		very			418	45.80		
		moderate			302	33.10		
		little			141	15.40		
		not at all			52	5.70		
	Check-in procedures for staff							
		very			399	43.70		
		moderate			305	33.40		
		little			142	15.60		
		not at all			67	7.30		
